# Supplementary material for: Urinary Dickkopf 3 Is Not an Independent Risk Factor in a Cohort of Kidney Transplant Recipients and Living Donors
Source: Int J Mol Sci. 2024 May 15;25(10):5376. doi: 10.3390/ijms25105376 (PMC11121870; doi:10.3390/ijms25105376)
Supplement: Supplementary file 1 [file ijms-25-05376-s001.zip › ijms-2940377-supplementary.pdf]

Supplementary Materials:

Suppl. Figure S1:

A

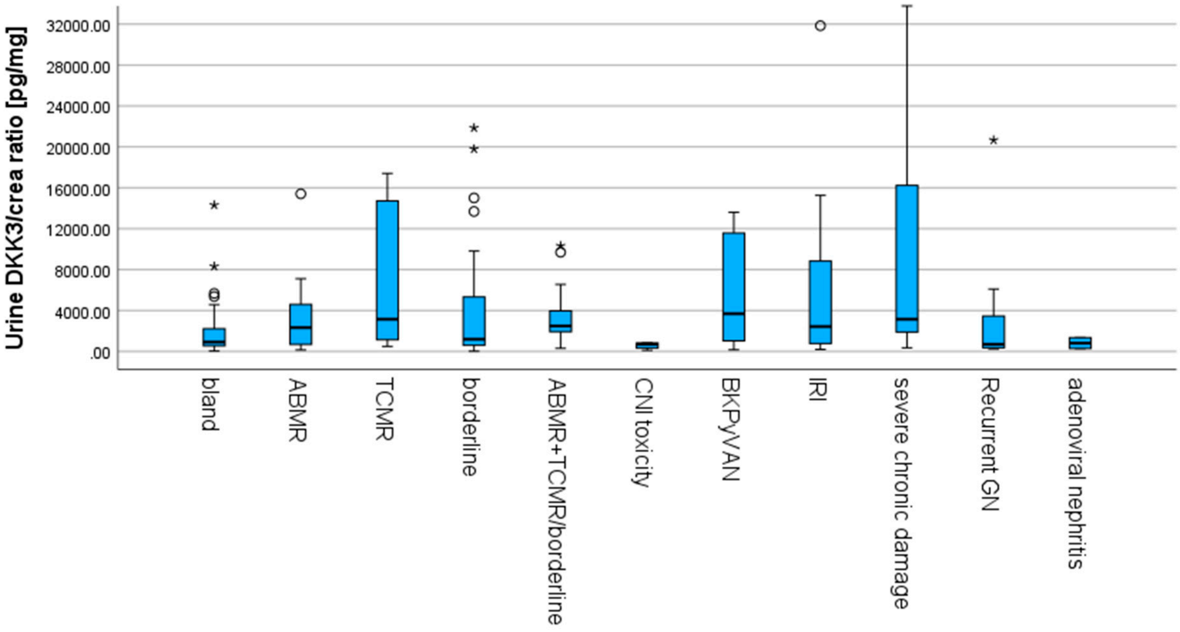

B

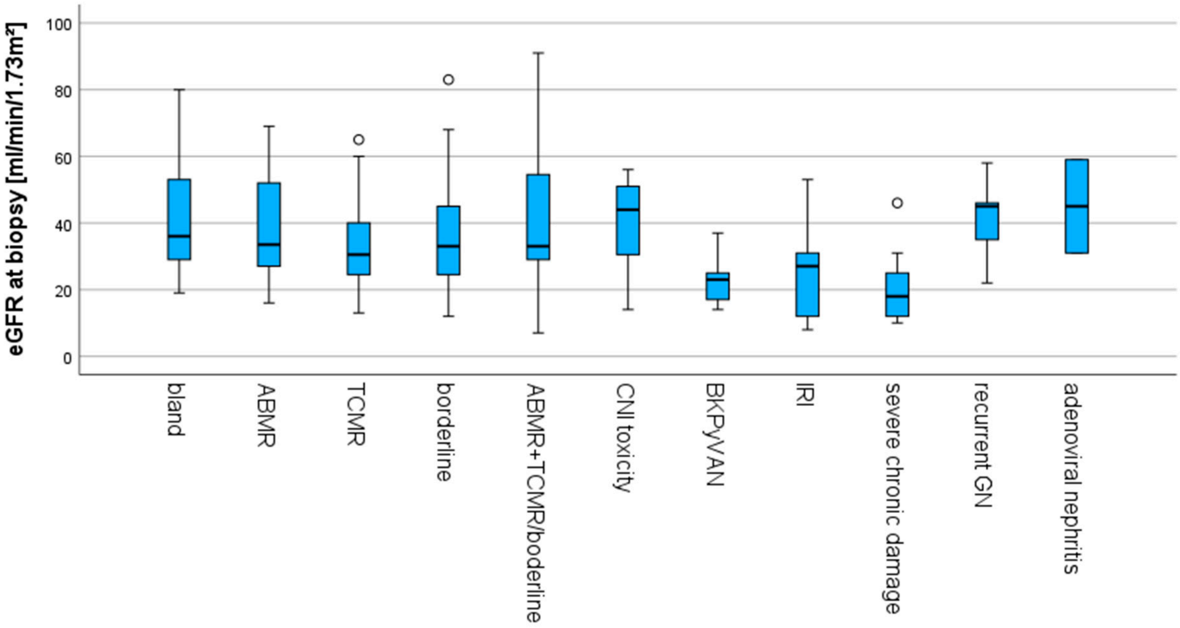

Suppl. Figure S1: uDKK3/Crea ratio values separated for the different histologic diagnoses are shown separately as box plots **(A).** The corresponding eGFR are also shown as box plots **(B)**.

The asterisk (*) marks an extreme value. The circle (o) represents an outlier.

Abbreviations: ABMR Antibody-mediated rejection; TCMR T-cell mediated rejection; CNI Calcineurin-inhibitor; BKPyVAN BK-Polyomavirus associated nephropathy; IRI ischemia-reperfusion injury; GN Glomerulonephritis

Suppl. Figure S2:

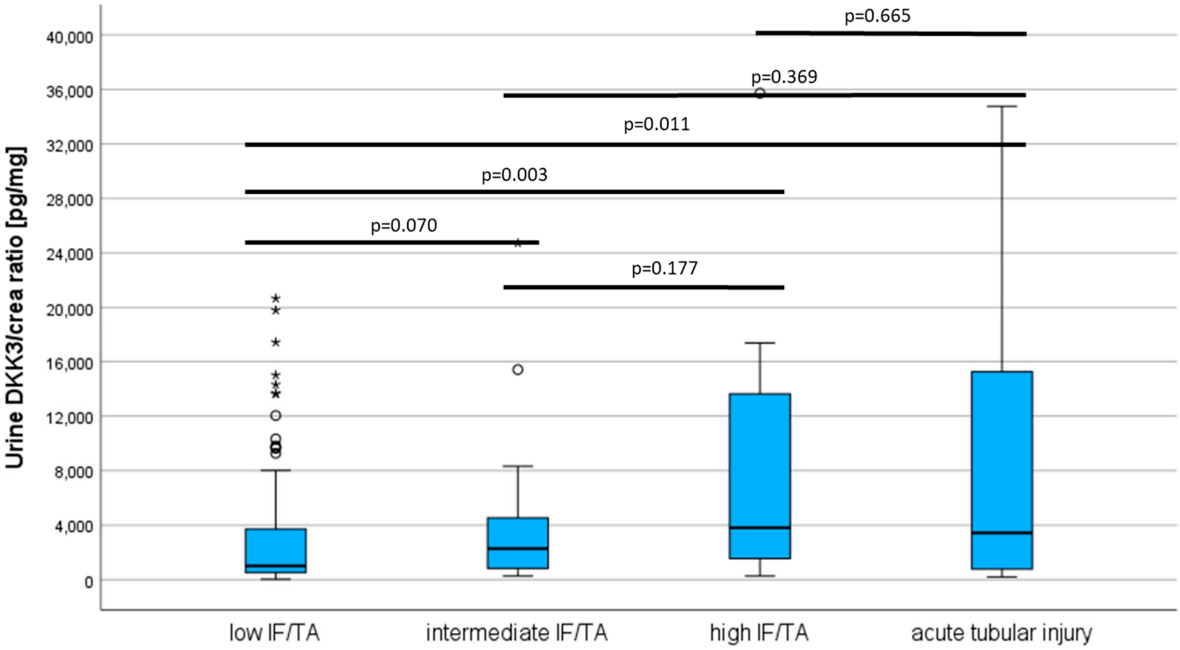

Suppl. Figure S2: uDKK3/Crea ratio levels separated for the different histologic diagnoses are shown separately as box plots.

The asterisk (*) marks an extreme value. The circle (o) represents an outlier.

Suppl. Table S1: Multivariable linear regression analysis for the dependent variable *uDKK/crea ratio on day of biopsy.*

Variable	Regression-coefficient	95% CI	Beta	p value
**Histological diagnosis**	257.9	-441.9 – 957.7	**0.054**	**0.468**
**eGFR at biopsy day**	-264.1	-397.9 – 130.3	**-0.289**	**<0.001**

eGFR: estimated glomerula filtration rate, calculated by CKD-EPI formula.

Suppl. Table S2: Multinominal logistic regression analysis for the dependent variable *IF/TA, reference category low IF/TA*

	Variable	Regression-coefficient	95% CI	p value
vs. intermediate IF/TA	**uDKK3/crea ratio at biopsy day**	1.000	0.992 – 1.008	**0.995**
	**eGFR at biopsy day**	0.975	0.949 – 1.003	**0.075**
vs. high IF/TA	**uDKK3/crea ratio at biopsy day**	1.006	0.999 – 1.013	**0.082**
	**eGFR at biopsy day**	0.907	0.857 – 0.960	**<0.001**

IF/TA: interstitial fibrosis and tubular atrophy; uDKK3: urinary dickkopf3; crea: creatinine; eGFR: estimated glomerula filtration rate, calculated by CKD-EPI formula

Suppl. Table S3. Multivariable cox regression analysis for the incidence of definite allograft failure.

Variable	Regression-coefficient	95% CI	p value
**uDKK3/crea ratio on biopsy day**	0.999	0.996 – 1.001	**0.257**
**eGFR on biopsy day**	0.936	0.911 – 0.962	**<0.001**

uDKK3: urinary dickkopf3; crea: creatinine; eGFR: estimated glomerula filtration rate, calculated by CKD-EPI formula.
